# Supplementary material for: Cryptoasset networks: Flows and regular players in Bitcoin and XRP
Source: PLoS One. 2022 Aug 22;17(8):e0273068. doi: 10.1371/journal.pone.0273068 (PMC9394853; doi:10.1371/journal.pone.0273068)
Supplement: S1 File — (PDF) [file pone.0273068.s001.pdf]

Supporting Information

Cryptoasset networks: Flows and regular players in Bitcoin and XRP

Hideaki Aoyama[1,2,3], Yoshi Fujiwara[4], Yoshimasa Hidaka[5,6,2], Yuichi Ikeda[1]

1 Graduate School of Advanced Integrated Studies in Human Survivability, Kyoto University, Kyoto 606-8306, Japan
2 RIKEN iTHEMS, Wako 351-0198, Japan
3 Research Institute of Economy, Trade and Industry, Tokyo 100-0013, Japan
4 Graduate School of Information Science, University of Hyogo, Kobe 650-0047, Japan
5 KEK Theory Center, Tsukuba 305-0801, Japan
6 Graduate University for Advanced Studies (Sokendai), Tsukuba 305-0801, Japan

Appendix A Granger Causality Analysis of Price and Number of Players

Our VAR (vector auto regressive) model describes the time evolution of the log change rate of the price of cryptoassets, the number of IN players, and the number of BAL players. In the following, we denote the log change rate of the price of cryptoasset by y_t and the log change rate of the number of IN players and BAL players by x_t. As the evaluation criterion for the VAR model, we use the mean square error

$$M[y_t|\mathcal{U}] = E[(y_t - \hat{y}_t)^2], \tag{S1}$$

where $\mathcal{U}$ is the information used in the model and $\hat{y}_t$ is the prediction by the model defined by

$$\hat{y}_t = E[y_t|\mathcal{U}]. \tag{S2}$$

In Granger causality analysis, x_t is interpreted to be the cause of y_t when the following relation

$$M[y_t|\mathcal{X}(t-1) \cup \mathcal{Y}(t-1)] < M[y_t|\mathcal{Y}(t-1)] \tag{S3}$$

is satisfied. It also interprets x_t as being the instantaneous cause of y_t when the relation

$$M[y_t|x_t \cup \mathcal{X}(t-1) \cup \mathcal{Y}(t-1)] < M[y_t|\mathcal{X}(t-1) \cup \mathcal{Y}(t-1)] \tag{S4}$$

is satisfied. Here $\mathcal{X}(t-1)$ is the information set about past x_t, and $\mathcal{Y}(t-1)$ is the information set about past y_t.

Granger causality analysis requires a stationary time series process. We conducted a unit root test to determine if it is a stationary process. The obtained augmented Dickey–Fuller statistic and the p-values are shown in Tables S1 and S2. The p-values were less than 5% for all variables and periods except where underlined. These results confirm that the overall process is stationary.

We performed a Granger causality analysis to examine the significance of the effect of changes in the number of players x_t on price changes y_t. Tables S3, S4, S5, and S6 show the F statistic and the p-value, which is the probability of hitting a value greater than the F statistic in an F distribution with two degrees of freedom df_1 and df_2 for active IN player, active BAL player, regular IN player, and regular BAL player, respectively, obtained in the Granger causality analysis. For all variables and periods, the p-values are very large. These results indicated in the tables mean that x_t is not interpreted to be the cause of y_t for all cases.

In addition, We performed an instantaneous causality analysis to examine the significance of the changes in the number of players x_t as being the instantaneous cause of price changes y_t. Tables S7, S8, S9, and S10 show the χ^2 value and the p-value, which is the probability of hitting a value greater than the χ^2 value in an χ^2 distribution with degree of freedom df for active IN player, active BAL player, regular IN player, and regular BAL player, respectively, obtained in the instantaneous causality analysis. The p-values show sufficiently small values for all variables and periods except where underlined. These results indicated in the tables mean that the x_t is interpreted to be the instantaneous cause of y_t for all cases.

In summary, x_t is not the cause of y_t in the Granger sense. Including x_t in the model does not improve the prediction accuracy of y_t. On the other hand, x_t

is a instantaneous cause of y_t. However, instantaneous causality does not lead to more accurate forecasts. These results indicate that it is difficult to forecast the prices of cryptoasset using the number of players. These results support the results discussed in Table 5, Table 6, and Table 7.

Table S1: Unit root test of the return and the active player

	Return		active IN		active BAL	
Period	ADF	p-value	ADF	p-value	ADF	p-value
2016-07-03 to 2017-07-01	-5.22	0.00064	-3.43	0.062	-7.52	4.3e-07
2017-07-02 to 2018-06-30	-4.53	0.0043	-2.52	0.32	-5.16	0.00077
2018-07-01 to 2019-06-29	-4.09	0.013	-5.01	0.0012	-2.75	0.22
2019-06-30 to 2020-06-27	-4.92	0.0015	-6.26	3.1e-05	-4.4	0.0061
2020-06-28 to 2021-06-26	-4.45	0.0052	-6.92	3.7e-06	-6.59	1.1e-05

Table S2: Unit root test of the regular player

	regular IN		regular BAL	
Period	ADF	p-value	ADF	p-value
2016-07-03 to 2017-07-01	-6.25	3.2e-05	-6.14	4.5e-05
2017-07-02 to 2018-06-30	-3.3	0.081	-6.57	1.2e-05
2018-07-01 to 2019-06-29	-6.13	4.6e-05	-5.86	1e-04
2019-06-30 to 2020-06-27	-4.46	0.0052	-4.73	0.0025
2020-06-28 to 2021-06-26	-5	0.0012	-6.01	6.6e-05

Table S3: Granger causality of the active IN player

Period	F-statistic	df_1	df_2	p-value
2016-07-03 to 2017-07-01	0.515	4	76	0.72
2017-07-02 to 2018-06-30	0.956	4	76	0.44
2018-07-01 to 2019-06-29	0.568	4	76	0.69
2019-06-30 to 2020-06-27	0.251	4	76	0.91
2020-06-28 to 2021-06-26	0.811	4	76	0.52

Table S4: Granger causality of the active BAL player

Period	F-statistic	df_1	df_2	p-value
2016-07-03 to 2017-07-01	0.515	4	76	0.72
2017-07-02 to 2018-06-30	0.956	4	76	0.44
2018-07-01 to 2019-06-29	0.568	4	76	0.69
2019-06-30 to 2020-06-27	0.251	4	76	0.91
2020-06-28 to 2021-06-26	0.811	4	76	0.52

Table S5: Granger causality of the regular IN player

Period	F-statistic	df_1	df_2	p-value
2016-07-03 to 2017-07-01	0.614	4	76	0.65
2017-07-02 to 2018-06-30	0.378	4	76	0.82
2018-07-01 to 2019-06-29	0.968	4	76	0.43
2019-06-30 to 2020-06-27	2.54	4	76	0.046
2020-06-28 to 2021-06-26	1.35	4	76	0.26

Table S6: Granger causality of the regular BAL player

Period	F-statistic	df_1	df_2	p-value
2016-07-03 to 2017-07-01	0.614	4	76	0.65
2017-07-02 to 2018-06-30	0.378	4	76	0.82
2018-07-01 to 2019-06-29	0.968	4	76	0.43
2019-06-30 to 2020-06-27	2.54	4	76	0.046
2020-06-28 to 2021-06-26	1.35	4	76	0.26

Table S7: Instantaneous causality of the active IN player

Period	χ^2	df	p-value
2016-07-03 to 2017-07-01	3.03	1	0.082
2017-07-02 to 2018-06-30	12.3	1	0.00046
2018-07-01 to 2019-06-29	7.04	1	0.008
2019-06-30 to 2020-06-27	0.174	1	<u>0.68</u>
2020-06-28 to 2021-06-26	15.4	1	8.6e-05

Table S8: Instantaneous causality of the active BAL player

Period	χ^2	df	p-value
2016-07-03 to 2017-07-01	3.03	1	0.082
2017-07-02 to 2018-06-30	12.3	1	0.00046
2018-07-01 to 2019-06-29	7.04	1	0.008
2019-06-30 to 2020-06-27	0.174	1	<u>0.68</u>
2020-06-28 to 2021-06-26	15.4	1	8.6e-05

Table S9: Instantaneous causality of the regular IN player

Period	χ^2	*df*	p-value
2016-07-03 to 2017-07-01	9.91	1	0.0016
2017-07-02 to 2018-06-30	3.46	1	0.063
2018-07-01 to 2019-06-29	11.7	1	0.00062
2019-06-30 to 2020-06-27	6.73	1	0.0095
2020-06-28 to 2021-06-26	4.26	1	0.039

Table S10: Instantaneous causality of the regular BAL player

Period	χ^2	*df*	p-value
2016-07-03 to 2017-07-01	9.91	1	0.0016
2017-07-02 to 2018-06-30	3.46	1	0.063
2018-07-01 to 2019-06-29	11.7	1	0.00062
2019-06-30 to 2020-06-27	6.73	1	0.0095
2020-06-28 to 2021-06-26	4.26	1	0.039

Appendix B Identity of Bitcoin Users

This supplement explains how we obtain the identity and classification of business activities of regular playes for Bitcoin.

In the case of Bitcoin, we employed a simple but useful method, proposed by [1] and widely used in the literature, to identify users from wallets or addresses by identifying a set of multiple input of addresses in each transaction as a user in the way that the identification must be consistent in the entire history of all the transactions up to a certain point in time (see [2] for technical details). Our study is based on this method and on the entire history from the genesis block (first block issued on January 9, 2009) until the block of height 693,999 (issued on August 3, 2021).

As a result, among more than 700 million different addresses, 500 million of them were identified as 73 million users. A user corresponds to two or more addresses by the above mentioned method of identification. A user can possess a large number of addresses; the largest one corresponds to the case of 13 million addresses for a single user. We denote such users with two or more addresses by *type A*. The rest of unidentified addresses are regarded as users individually, denoted by *type B*. We label a user of type A by a user ID, sequential and increasing with the number of identified addresses, e.g. `0000012345`, while a user of type B is labeled by its address, e.g. `3CjqmbuRA1LEWmLHiWoSWHcWuTEVPfU24P`.

Because of the very nature of anonymity inherent in the technology of blockchain, it is difficult to obtain the information of actual names of those users. However, in the case of agents who are doing business activities such as exchanges, services, gambling, and mining, it is known that one can obtain the identify of users. Such information could be useful for our study, even if not complete and exhaustive, as we found in our paper. In fact, `WalletExplorer.com` [3] is a well known web site providing information about identity of addresses in Bitcoin blockchain. The site merges addresses together, if they are part of the same wallet, and also identifies wallets with actual names. According to the site, the method to merge addresses is precisely the same as [1], which is the one we employed in our paper. Additionally, the identification of actual names is done by `WalletExplorer.com` as follows (quoted from the web site):

> *In most of the cases, I registered to service, made transaction(s) and saw which wallet bitcoins were merged with, or from which wallet it was withdrawn.*
> *There is probably no easier way how to discover names other than this.*
> *Please note that the name database is not updated, so it does not contain newer exchanges (or newer wallets of existing exchanges).*

We matched our data with this useful information to obtain the identity and classification of business activities for the users of type A. We were successful in unravel the identity of 369 users in this way, which are used in the main body of our paper. Table S11 is the classification into exchanges, services, gambling, historic, and mining pools. Table S12 is the complete list of the matching.

Table S11: Classification of identified users (compiled from [3])

Classification	#Users	Examples
Exchanges	116	`Bittrex.com`, `Huobi.com`, `Bit-x.com`, `HitBtc.c om`
Old/Historic	94	`AgoraMarket`, `EvolutionMarket`, `SilkRoadMarketplace`
Services/Others	89	`Xapo.com`, `ePay.info`, `Cubits.com`
Gambling	52	`999Dice.com`, `CoinGaming.io`, `SatoshiMines.com`
Pools	18	`BTCCPool`, `SlushPool.com`, `BitMinter.com`
Total	369	—

Table S12: Identity of Users (compiled from [3])

Definitions
No: sequential number
User ID: an arbitrarily but uniquely assigned IDs to each user in our data
#Addr.: number of addresses identified to each User ID in our data (rows are sorted by this column)
Name: Web site (N.B. `old`, `hotwallet`, etc. are additional information)
Category: Exchanges, Old/Historic, Services/Others, Gambling, Pools

No.	User ID	#Addr.	Name	Category
1	0000000001	13,206,603	Bitcoin-24.com-old-hotwallet	Old/Historic
2	0000000001	13,206,603	ePay.info	Services/Others
3	0000000002	9,263,042	CoinPayments.net	Services/Others
4	0000000005	2,183,405	Xapo.com	Services/Others
5	0000000013	1,636,640	Bittrex.com	Exchanges
6	0000000014	1,630,483	Cubits.com	Services/Others
7	0000000016	1,284,661	Kraken.com	Exchanges
8	0000000018	1,195,265	Huobi.com-2	Exchanges
9	0000000022	1,019,378	Poloniex.com	Exchanges
10	0000000029	962,592	Cryptonator.com	Services/Others
11	0000000033	912,950	AnxPro.com	Exchanges
12	0000000033	912,950	CoinTrader.net	Exchanges
13	0000000033	912,950	LocalBitcoins.com-old	Exchanges
14	0000000036	879,110	Luno.com	Exchanges
15	0000000042	810,136	999Dice.com	Gambling
16	0000000053	659,719	CoinGaming.io	Gambling
17	0000000063	523,330	LocalBitcoins.com	Exchanges
18	0000000065	498,001	AgoraMarket	Old/Historic
19	0000000069	478,854	Bitstamp.net	Exchanges
20	0000000079	420,632	EvolutionMarket	Old/Historic
21	0000000086	372,753	SilkRoadMarketplace	Old/Historic
22	0000000087	369,593	MercadoBitcoin.com.br	Exchanges
23	0000000091	350,036	SilkRoad2Market	Old/Historic
24	0000000093	348,491	BTC-e.com-old	Exchanges
25	0000000108	307,508	BTC-e.com	Exchanges
26	0000000111	287,684	VIP72.com	Services/Others
27	0000000114	280,963	Bitcoin.de	Exchanges
28	0000000120	268,458	SatoshiMines.com	Gambling
29	0000000123	263,074	YoBit.net	Exchanges
30	0000000124	259,401	Cryptsy.com-old	Exchanges
31	0000000125	249,048	Binance.com	Exchanges
32	0000000126	244,678	BitcoinFog	Services/Others
33	0000000133	238,480	Cex.io	Exchanges
34	0000000144	207,477	CoinJar.com	Services/Others
35	0000000145	205,601	BitZlato.com	Exchanges
36	0000000152	197,164	NitrogenSports.eu	Gambling
37	0000000159	189,776	AlphaBayMarket	Services/Others
38	0000000162	187,189	HitBtc.com	Exchanges
39	0000000165	186,000	BitPay.com	Services/Others
40	0000000187	172,413	BitPay.com-old2	Services/Others
41	0000000198	166,077	BitPay.com-old	Services/Others
42	0000000203	161,974	BitPay.com-old3	Services/Others
43	0000000221	146,381	NucleusMarket	Services/Others
44	0000000234	138,182	Bitstamp.net-old	Exchanges

Continue to next page

Table S12 continued from previous page

No.	User ID	#Addr.	Name	Category
45	0000000239	134,562	Cryptsy.com	Exchanges
46	0000000241	134,257	CloudBet.com	Gambling
47	0000000258	125,004	PocketDice.io	Gambling
48	0000000265	120,562	FortuneJack.com	Gambling
49	0000000266	119,119	AbraxasMarket	Old/Historic
50	0000000276	115,775	CoinKite.com	Services/Others
51	0000000277	114,999	Bleutrade.com	Exchanges
52	0000000279	114,464	Kraken.com-old	Exchanges
53	0000000284	109,151	Instawallet.org	Old/Historic
54	0000000303	97,347	Bitcoin.de-old	Exchanges
55	0000000305	96,890	SecondsTrade.com	Gambling
56	0000000322	87,705	BitcoinWallet.com	Services/Others
57	0000000323	87,424	BitoEX.com	Services/Others
58	0000000326	85,626	MintPal.com	Old/Historic
59	0000000328	85,590	Hashnest.com	Exchanges
60	0000000335	83,724	BtcTrade.com	Exchanges
61	0000000343	80,987	OKCoin.com-2	Exchanges
62	0000000347	79,712	Bter.com	Exchanges
63	0000000352	78,849	BitZino.com	Gambling
64	0000000354	78,119	OKCoin.com	Exchanges
65	0000000355	77,666	Bitfinex.com-old2	Exchanges
66	0000000363	74,602	Rollin.io	Gambling
67	0000000387	68,927	VirWoX.com	Exchanges
68	0000000414	64,803	BTCC.com	Exchanges
69	0000000451	57,770	MaiCoin.com	Exchanges
70	0000000456	56,953	BTCCPool	Pools
71	0000000465	55,757	PandoraOpenMarket	Old/Historic
72	0000000471	55,271	Paxful.com	Exchanges
73	0000000474	54,640	PrimeDice.com	Gambling
74	0000000480	53,775	PrimeDice.com-old4	Gambling
75	0000000482	53,639	SheepMarketplace	Old/Historic
76	0000000486	53,102	Cavirtex.com	Exchanges
77	0000000501	51,039	MoonBit.co.in	Services/Others
78	0000000503	50,878	BlackBankMarket	Old/Historic
79	0000000507	50,295	HaoBTC.com	Services/Others
80	0000000517	48,602	BX.in.th	Exchanges
81	0000000530	47,295	Matbea.com	Exchanges
82	0000000548	46,051	PrimeDice.com-old3	Gambling
83	0000000565	44,277	CoinMotion.com	Exchanges
84	0000000585	42,781	SatoshiDice.com	Gambling
85	0000000591	42,301	PrimeDice.com-old2	Gambling
86	0000000611	41,156	BTCJam.com	Services/Others
87	0000000665	36,999	Justcoin.com	Old/Historic
88	0000000679	35,545	SafeDice.com	Gambling
89	0000000682	35,495	BTCC.com-old	Exchanges
90	0000000683	35,453	McxNOW.com	Old/Historic
91	0000000684	35,433	C-Cex.com	Exchanges
92	0000000706	34,149	MiddleEarthMarketplace	Old/Historic
93	0000000714	33,436	Vircurex.com	Exchanges
94	0000000724	32,823	Purse.io	Services/Others
95	0000000726	32,701	SatoshiBet.com	Gambling
96	0000000737	32,017	SwCPoker.eu	Gambling
97	0000000739	31,940	BitBargain.co.uk	Exchanges
98	0000000753	30,965	SealsWithClubs.eu	Old/Historic
99	0000000757	30,704	CoinHako.com	Exchanges
100	0000000761	30,187	OkLink.com	Services/Others
101	0000000772	29,256	Huobi.com	Exchanges
102	0000000796	27,892	Bit-x.com	Exchanges
103	0000000823	26,643	BtcDice.com	Old/Historic
104	0000000838	26,014	BitBay.net	Exchanges
105	0000000839	25,960	Betcoin.ag	Gambling
106	0000000868	24,445	Bter.com-old	Exchanges
107	0000000880	23,886	Paymium.com	Services/Others
108	0000000882	23,833	BTCC.com-old2	Exchanges
109	0000000912	22,597	Loanbase.com	Services/Others
110	0000000916	22,304	Coinroll.com	Gambling
111	0000000930	21,693	FaucetBOX.com	Services/Others
112	0000000940	21,339	BitcoinVideoCasino.com-old	Gambling
113	0000000960	20,730	CampBX.com-old	Exchanges
114	0000000969	20,502	FYBSG.com	Exchanges

Continue to next page

Table S12 continued from previous page

No.	User ID	#Addr.	Name	Category
115	0000000998	19,810	TheRockTrading.com	Exchanges
116	0000001025	18,997	BlueSkyMarketplace	Old/Historic
117	0000001035	18,489	Crypto-Games.net	Gambling
118	0000001066	17,705	Coin-Swap.net	Old/Historic
119	0000001084	17,365	BitcoinVideoCasino.com-old2	Gambling
120	0000001097	17,137	TheRockTrading.com-old	Exchanges
121	0000001150	15,965	AnoniBet.com	Gambling
122	0000001155	15,905	ChangeTip.com	Services/Others
123	0000001162	15,757	Bitmit.net	Old/Historic
124	0000001168	15,495	CoinApult.com	Services/Others
125	0000001182	15,260	BtcMarkets.net	Exchanges
126	0000001213	14,566	Inputs.io	Old/Historic
127	0000001228	14,233	CrimeNetwork.co	Services/Others
128	0000001243	13,972	Bter.com-old2	Exchanges
129	0000001258	13,713	Vaultoro.com	Exchanges
130	0000001326	12,486	CryptoStocks.com	Services/Others
131	0000001328	12,456	BitAces.me	Old/Historic
132	0000001343	12,118	HelixMixer-old18	Services/Others
133	0000001418	11,221	Coins-e.com	Exchanges
134	0000001419	11,220	Igot.com	Exchanges
135	0000001444	10,901	SatoshiRoulette.com	Gambling
136	0000001447	10,837	HelixMixer-old34	Services/Others
137	0000001469	10,601	HelixMixer-old33	Services/Others
138	0000001484	10,357	HelixMixer-old16	Services/Others
139	0000001499	10,264	HelixMixer-old27	Services/Others
140	0000001508	10,195	Bter.com-old3	Exchanges
141	0000001564	9,666	HelixMixer-old4	Services/Others
142	0000001591	9,512	Crypto-Trade.com	Old/Historic
143	0000001604	9,430	HelixMixer-old28	Services/Others
144	0000001605	9,428	CoinVault	Old/Historic
145	0000001622	9,274	HelixMixer-old19	Services/Others
146	0000001628	9,210	SlushPool.com	Pools
147	0000001634	9,165	Cryptorush.in	Old/Historic
148	0000001641	9,122	BTCOracle.com	Gambling
149	0000001662	8,967	Genesis-Mining.com	Services/Others
150	0000001675	8,882	HelixMixer-old2	Services/Others
151	0000001692	8,767	HelixMixer-old14	Services/Others
152	0000001693	8,751	HelixMixer-old20	Services/Others
153	0000001734	8,444	HelixMixer-old17	Services/Others
154	0000001736	8,430	Exmo.com	Exchanges
155	0000001755	8,370	HelixMixer-old9	Services/Others
156	0000001787	8,120	VaultOfSatoshi.com	Old/Historic
157	0000001807	8,032	BitcoinVideoCasino.com	Gambling
158	0000001809	8,030	PrimeDice.com-old	Gambling
159	0000001814	8,006	HelixMixer-old12	Services/Others
160	0000001821	7,977	HelixMixer-old15	Services/Others
161	0000001834	7,865	BTCGuild.com	Old/Historic
162	0000001836	7,857	Peerbet.org	Gambling
163	0000001839	7,848	796.com	Exchanges
164	0000001866	7,605	HelixMixer-old10	Services/Others
165	0000001867	7,603	HitBtc.com-old	Exchanges
166	0000001869	7,585	Btc38.com	Exchanges
167	0000001889	7,479	Betcoins.net	Old/Historic
168	0000001920	7,302	HelixMixer-old21	Services/Others
169	0000001946	7,172	HelixMixer-old23	Services/Others
170	0000001952	7,151	AlphaBayMarket-old	Services/Others
171	0000001967	7,109	LiteBit.eu	Exchanges
172	0000001972	7,088	HelixMixer-old25	Services/Others
173	0000002027	6,853	HelixMixer-old30	Services/Others
174	0000002043	6,790	Bitbond.com	Services/Others
175	0000002053	6,747	HappyCoins.com	Exchanges
176	0000002059	6,736	HelixMixer-old13	Services/Others
177	0000002122	6,477	Bitcoin-Roulette.com	Old/Historic
178	0000002151	6,356	HelixMixer-old7	Services/Others
179	0000002163	6,309	AllCoin.com	Old/Historic
180	0000002185	6,255	HelixMixer-old24	Services/Others
181	0000002187	6,242	Coin.mx	Old/Historic
182	0000002205	6,179	HelixMixer-old3	Services/Others
183	0000002235	6,046	LakeBTC.com	Exchanges
184	0000002238	6,019	HelixMixer-old6	Services/Others

Continue to next page

Table S12 continued from previous page

No.	User ID	#Addr.	Name	Category
185	0000002259	5,966	777Coin.com	Gambling
186	0000002273	5,934	GHash.io	Pools
187	0000002279	5,907	Bitcoin-24.com-old	Old/Historic
188	0000002297	5,840	CrimeNetwork.co-old	Services/Others
189	0000002319	5,762	DoctorDMarket	Services/Others
190	0000002379	5,576	C-Cex.com-old	Exchanges
191	0000002403	5,483	HelixMixer-old22	Services/Others
192	0000002404	5,481	Coinomat.com	Exchanges
193	0000002474	5,297	Coinmate.io	Exchanges
194	0000002495	5,253	HelixMixer-old31	Services/Others
195	0000002518	5,188	HelixMixer-old26	Services/Others
196	0000002586	5,035	HelixMixer-old29	Services/Others
197	0000002591	5,024	BitVC.com	Exchanges
198	0000002607	4,996	SatoshiCircle.com	Gambling
199	0000002647	4,896	MyBitcoin.com	Old/Historic
200	0000002698	4,775	AllCrypt.com	Old/Historic
201	0000002761	4,629	GermanPlazaMarket	Services/Others
202	0000002776	4,605	MasterXchange.com	Old/Historic
203	0000002792	4,560	CoinCafe.com	Exchanges
204	0000002808	4,530	BitKonan.com	Exchanges
205	0000002814	4,517	QuadrigaCX.com	Exchanges
206	0000002836	4,451	BitElfin.com	Old/Historic
207	0000002844	4,435	SpectroCoin.com	Exchanges
208	0000002871	4,377	OrderBook.net	Exchanges
209	0000002884	4,358	Betcoin.ag-old	Gambling
210	0000002887	4,354	Bitcurex.com	Exchanges
211	0000002888	4,352	BitAces.me-old	Old/Historic
212	0000002893	4,338	Coinichiwa.com	Gambling
213	0000002915	4,292	Betcoin.tm	Gambling
214	0000002950	4,232	MeXBT.com	Exchanges
215	0000003070	3,999	Bitfinex.com	Exchanges
216	0000003109	3,937	HelixMixer-old32	Services/Others
217	0000003157	3,861	BetsOfBitco.in	Old/Historic
218	0000003167	3,840	JetWin.com	Gambling
219	0000003305	3,609	BitZillions.com	Gambling
220	0000003349	3,545	Korbit.co.kr	Exchanges
221	0000003403	3,485	BTCPop.co	Services/Others
222	0000003515	3,356	HelixMixer-old5	Services/Others
223	0000003550	3,308	BTC-e.com-output	Exchanges
224	0000003617	3,238	YABTCL.com	Gambling
225	0000003716	3,121	BIToomBa.com	Old/Historic
226	0000003752	3,086	BitYes.com	Old/Historic
227	0000003770	3,065	FoxBit.com.br-cold	Exchanges
228	0000003834	2,998	CoinURL.com	Services/Others
229	0000003972	2,848	HelixMixer-old8	Services/Others
230	0000003985	2,829	CannabisRoadMarket	Old/Historic
231	0000004072	2,760	Ice-Dice.com	Old/Historic
232	0000004094	2,744	ChBtc.com	Exchanges
233	0000004122	2,724	Bitfinex.com-old	Exchanges
234	0000004135	2,713	CoinArch.com	Exchanges
235	0000004198	2,652	Banx.io-old	Exchanges
236	0000004205	2,645	Comkort.com	Old/Historic
237	0000004214	2,636	Bitcoinica.com-old	Old/Historic
238	0000004247	2,618	BitNZ.com	Services/Others
239	0000004255	2,614	CleverCoin.com	Exchanges
240	0000004309	2,575	CoinMkt.com	Old/Historic
241	0000004457	2,459	HelixMixer-old	Services/Others
242	0000004609	2,355	DiceBitco.in	Old/Historic
243	0000004747	2,276	BitcoinVietnam.com.vn	Exchanges
244	0000004851	2,221	Indacoin.com	Exchanges
245	0000005248	2,023	BitClix.com	Services/Others
246	0000005320	1,992	Coin-Sweeper.com	Old/Historic
247	0000005426	1,948	GoCelery.com	Services/Others
248	0000005438	1,941	Satoshi-Karoshi.com-old	Gambling
249	0000005799	1,812	Playt.in	Old/Historic
250	0000005811	1,805	HelixMixer-old11	Services/Others
251	0000005814	1,804	Bitcash.cz	Old/Historic
252	0000005823	1,802	CampBX.com	Exchanges
253	0000006116	1,713	BTCLend.org	Services/Others
254	0000006145	1,704	CoinChimp.com	Exchanges

Continue to next page

Table S12 continued from previous page

No.	User ID	#Addr.	Name	Category
255	0000006170	1,699	BtcExchange.ro	Old/Historic
256	0000006199	1,690	AdmiralCoin.com	Old/Historic
257	0000006222	1,681	Cryptopay.me-old	Services/Others
258	0000006351	1,643	Bitcoinica.com	Old/Historic
259	0000006425	1,623	Coingi.com	Exchanges
260	0000006514	1,594	Gatecoin.com	Exchanges
261	0000006610	1,572	Bter.com-cold	Exchanges
262	0000006854	1,508	BetChain.com-old	Gambling
263	0000007050	1,471	BabylonMarket	Old/Historic
264	0000007174	1,443	HelixMixer	Services/Others
265	0000007356	1,401	Cryptonator.com-old	Services/Others
266	0000007428	1,389	SlushPool.com-old2	Pools
267	0000007850	1,314	Bylls.com	Services/Others
268	0000008204	1,246	Btcst.com-pirateat40	Old/Historic
269	0000008226	1,244	BitcoinWeBank.com	Old/Historic
270	0000008600	1,189	PocketRocketsCasino.eu	Old/Historic
271	0000008605	1,188	Bitso.com	Exchanges
272	0000008652	1,181	CoinRoyale.com-old2	Gambling
273	0000008671	1,178	BTCt.com	Old/Historic
274	0000008682	1,176	DaDice.com	Old/Historic
275	0000008940	1,140	SatoshiDice.com-original	Gambling
276	0000009253	1,098	Cryptonit.net	Exchanges
277	0000009395	1,082	Dgex.com-old	Old/Historic
278	0000009446	1,076	BitStarz.com	Gambling
279	0000009819	1,040	Ccedk.com	Exchanges
280	0000010013	1,020	Satoshi-Karoshi.com	Gambling
281	0000010664	978	Just-Dice.com	Old/Historic
282	0000010770	968	CryptoLocker	Old/Historic
283	0000010798	965	GreenRoadMarket	Services/Others
284	0000010809	963	SlushPool.com-old	Pools
285	0000010941	953	CoinRoyale.com	Gambling
286	0000010985	950	CryptoBounty.com	Old/Historic
287	0000011403	915	Chainroll.com-old	Old/Historic
288	0000012399	846	CoinRoyale.com-old	Gambling
289	0000014332	741	EmpoEX.com	Exchanges
290	0000014593	729	FairProof.com	Gambling
291	0000015294	700	UseCryptos.com	Exchanges
292	0000016863	670	AntPool.com	Pools
293	0000016928	668	Coinbroker.io	Exchanges
294	0000017040	665	UpDown.BT	Old/Historic
295	0000017688	648	DiceNow.com	Gambling
296	0000017899	643	CrimeNetwork.cc-old	Old/Historic
297	0000017921	643	Dagensia.eu	Old/Historic
298	0000019081	614	WatchMyBit.com	Services/Others
299	0000019524	604	MPEx.co	Old/Historic
300	0000020410	593	Banx.io	Exchanges
301	0000020698	588	EclipseMC.com-old	Pools
302	0000021439	572	CloudHashing.com	Old/Historic
303	0000021756	565	Eligius.st	Pools
304	0000023664	527	Europex.eu	Old/Historic
305	0000024299	515	EveryDice.com	Old/Historic
306	0000024598	511	CrimeNetwork.cc	Old/Historic
307	0000025484	499	Brawker.com	Old/Historic
308	0000027631	473	10xBitco.in	Old/Historic
309	0000029250	450	Cryptonit.net-old	Exchanges
310	0000029949	440	BitMinter.com	Pools
311	0000033271	402	BW.com	Pools
312	0000033478	400	Chainroll.com	Old/Historic
313	0000035156	390	DiceCoin.io	Gambling
314	0000035978	382	FoxBit.com.br-2	Exchanges
315	0000036098	380	PonziCoin.co	Old/Historic
316	0000036395	377	Banx.io-old2	Exchanges
317	0000036405	377	Birwo.com-old	Old/Historic
318	0000039093	351	Zyado.com	Exchanges
319	0000040205	341	SuzukiDice.com	Old/Historic
320	0000043429	316	50BTC.com-old2	Old/Historic
321	0000047851	297	KnCMiner.com	Pools
322	0000048078	295	CrimeNetwork.biz	Old/Historic
323	0000048367	293	BitcoinPokerTables.com	Gambling
324	0000050538	280	Polmine.pl	Old/Historic

Continue to next page

Table S12 continued from previous page

No.	User ID	#Addr.	Name	Category
325	0000050829	279	MineField.BitcoinLab.org	Gambling
326	0000064447	239	Zyado.com-old	Exchanges
327	0000065935	234	SmenarnaBitcoin.cz	Old/Historic
328	0000072171	219	FoxBit.com.br-cold-old	Exchanges
329	0000077659	204	50BTC.com-old3	Old/Historic
330	0000089370	195	Dgex.com	Old/Historic
331	0000091528	190	BitLaunder.com	Services/Others
332	0000098744	176	AntPool.com-old2	Pools
333	0000102180	170	BitMillions.com	Old/Historic
334	0000133798	131	CrimeNetwork.cc-old3	Old/Historic
335	0000134052	131	CrimeNetwork.cc-old2	Old/Historic
336	0000140685	125	Vic-Socks.to	Services/Others
337	0000150036	117	Gatecoin.com-2	Exchanges
338	0000157956	112	Bitcoin-24.com	Old/Historic
339	0000217145	94	CoinApult.com-old	Services/Others
340	0000272989	77	BetcoinDice.tm	Old/Historic
341	0000278134	75	Bitfury.org	Pools
342	0000317097	66	BTradeAustralia.com-incoming	Exchanges
343	0000396933	53	50BTC.com	Old/Historic
344	0000478261	46	HolyTransaction.com	Services/Others
345	0000653557	35	SecureVPN.to	Services/Others
346	0000846261	28	DiceOnCrack.com	Old/Historic
347	0001226645	22	SecureVPN.to-old	Services/Others
348	0001385175	20	ActionCrypto.com	Old/Historic
349	0001443065	19	Kano.is-old	Pools
350	0001893594	15	ASICMiner	Old/Historic
351	0002158176	14	Telco214	Pools
352	0002496530	12	PinballCoin.com	Old/Historic
353	0002576310	12	BTradeAustralia.com	Exchanges
354	0002582950	12	SimpleCoin.cz-old4	Exchanges
355	0003951120	8	EclipseMC.com-old2	Pools
356	0004264612	8	SimpleCoin.cz-old2	Exchanges
357	0005131973	7	SimpleCoin.cz	Exchanges
358	0005465650	7	SimpleCoin.cz-old5	Exchanges
359	0007026550	6	LuckyB.it	Gambling
360	0007772443	5	Exchanging.ir	Exchanges
361	0010397557	4	FoxBit.com.br	Exchanges
362	0012987571	4	SimpleCoin.cz-old3	Exchanges
363	0013830337	4	EclipseMC.com-old3	Pools
364	0017583915	3	EclipseMC.com	Pools
365	0021692739	3	StrongCoin.com-fee	Services/Others
366	0027490563	2	DeepBit.net	Old/Historic
367	0029599951	2	BetMoose.com	Gambling
368	0043060825	2	Dispenser.tf	Old/Historic
369	0063718479	2	CoinWorker.com	Services/Others

End of Table S12

References

1. Reid F, Harrigan M. An Analysis of Anonymity in the Bitcoin System. In: Altshuler Y, Elovici Y, Cremers A, Aharony N, Pentland A, editors. Security and Privacy in Social Networks. Springer, New York; 2013. p. 197–223.

2. Antonopoulos AM. Mastering Bitcoin: Programming the Open Blockchain. 2nd ed. O'Reilly Media; 2017.

3. Bitcoin block explorer with address grouping and wallet labeling (accessed on 2022-03-29);. https://www.walletexplorer.com.
