# Supplementary material for: Cryptoasset networks: Flows and regular players in Bitcoin and XRP
Source: PLoS One. 2022 Aug 22;17(8):e0273068. doi: 10.1371/journal.pone.0273068 (PMC9394853; doi:10.1371/journal.pone.0273068)
Supplement: Attachment — Submitted filename: response.docx [file pone.0273068.s002.docx]

UNIVERSITY

OF HYOGO	**Prof. Yoshi Fujiwara, PhD**

Prof. Yoshi Fujiwara

*University of Hyogo*

Hocine Cherifi, Lead Guest Editor Editor-in-Chief

Reviewers PLOS ONE

July 22, 2022

*Kobe 650-0047, Japan*

*Email:* [*yoshi.fujiw*](mailto:yoshi.fujiwara@gmail.com)[*ara@gmail.com*](mailto:ara@gmail.com) *Phone: +81-783031976*

Dear Editors and Reviewers,

We would like to thank the editors and reviewers of our manuscript: *Hideaki Aoyama, Yoshi Fujiwara, Yoshimasa Hidaka, and Yuichi Ikeda, “Cryptoasset networks: Flows and regular players in Bitcoin and XRP”* as part of **as part of the Complex Networks Curated Collection - FAO: PLOS ONE PSE team**.

We are now resubmitting the revised version of our manuscript, which answers the points Reviewers raised. The response letter to the reviewers follows.

Yours Sincerely,

Prof. Yoshi Fujiwara, PhD

# Response to Reviewer 1

We thank the reviewer for the encouraging and thoughtful remarks and helpful suggestions. We have modified our manuscript in accordance to all of the raised points. Below please our explanation with the referee’s comments in italics and our responses in non-italics.

Please refer to the uploaded “Revised Manuscript with Track Changes” for the marked-up copy of our manuscript that highlights changes (in red) to the initial version, and also to the uploaded “Manuscript” for unmarked version of our revised paper without tracked changes.

## Main points

*Overall, this paper is well organized and contains some publishable material. However, two major problems exist. (1) Selecting the most significant period of one year as an observation period, it is doubtful whether the conclusions drawn are universal and representative.*

## response

We performed additional analysis for different epochs that cover several crypto bubbles and crashes in order to see how the cryptocurrency market’s changes affect the dynamics under our study. See the Discussion section "Comparison with other periods", and Figures 9 and 10 in addition to a systematic comparison by using correlations between price changes and the numbers of players. See Tables 5 and 6 for the results, also Table 7 for the other periods. Obviously we do not claim that the conclusions drawn are universal nor representative. Rather, by adding the above mentioned analysis on different periods and showing the results, we would like to show that our method of flow-weighted frequency and finding of three branches could potentially shed lights on how the players, especially regular players, behave under dramatically varying situations of bull or bear markets of cryptoassets.

(2) *the mathematical formula (11)–(13) are wrong and cannot achieve the purpose of dividing the set into three regions of 30 degrees.*

## response

It was a typo, our mistake, in the original manuscript as the reviewer kindly pointed out. We sincerely thank the reviewer, and corrected those equations so that they make sense for our purpose to classify the players into three regions of 30 degrees.

(3) *in the Results section, the variation between some weeks and the rest of the year is not distinct enough to draw a clear conclusion.*

## response

Presumably this point is related to the very first point above. We added a systematic comparison by using correlations between price changes and the numbers of players in the Results. See Tables 5 and 6, and main	texts.

*In addition, apart from the fact that the research objects are Bitcoin and XRP, it is hard to judge whether the differences between the research methods in this paper and existing methods are large enough to be publishable.*

## response

The flow-Frequency was developed by the current authors and it lead to the finding of the three-branch structures in Bitcoin and XRP transaction. In this paper, this finding was elaborated in detail for the first time and it was proven that it provides us with as new tool to classify the players and examine their behavior. The existence of this structure was never known before in any transaction networks and further application is being made currently to extend the application of the current method to other currency exchange networks, This paper marks an important basis for further, fruitful area of research, which was not possible with other existing methods.

# Response to Reviewer 2

We thank the reviewer for the encouraging and thoughtful remarks and helpful suggestions. We have modified our manuscript in accordance to all of the raised points. Below please read our explanation with the referee’s comments in italics and our responses in non-italics.

Please refer to the uploaded “Revised Manuscript with Track Changes” for the marked-up copy of our manuscript that highlights changes (in red) to the initial version, and also to the uploaded “Manuscript” for unmarked version of our revised paper without tracked changes.

## Comment

**Period under study**

*The analysis is quite attractive, but it covers only one particular example. The cryp- tocurrency market has changed and matured significantly since then - see for example https://doi.org/10.1016/j.physrep.2020.10.005, which makes this analysis his- torical. It will be really nice to see such kind of analysis for 2021 crypto bubble.*

## response

First, we performed additional analysis for different epochs that cover several crypto bubbles and crashes in order to see how the cryptocurrency market’s changes affect the dynamics under our study. See the Discussion section "Comparison with other periods", and Figures 9 and 10 in addition to a systematic comparison by using correlations between price changes and the numbers of players. See also the Additional comment 2 below.

Second, we would like to thank the reviewer for guiding us the reference, Marcin Wa¸ torek, Stanisław Drożdż, Jarosław Kwapień, Ludovico Minati, Paweł Oświe¸cimka and Marek Stanuszek, “Multiscale characteristics of the emerging global cryptocurrency market” published in Physics Reports (2021), which we were not aware of at the writing of initial manuscript. We added this reference in the Introduction section, so that the reader can access one of the most recent works relevant to our paper. Also the paper is cited at the Discussion section "Comparison with other periods". We sincerely thank the reviewer for the help.

## Additional comments

1. *To have a direct comparison of price changes with changes in the number of network users, price changes could be also added in Figs. 3 and	4.*

## response

We modified each of those figures so that the reader can directly do such a comparison. By doing so, we believe that it is easier for the reader to do the comparison by visual inspection.

2. *Did you try to calculate correlations between price changes or lagged price changes and ratio in/out in/bal out/bal players?*

## response

Thank you for pointing this out. In the revised manuscript, we have done additional analysis precisely for this purpose, and added the correlation between the time difference of lagged price, ∆*p*(*t k*), and the time difference of the numbers of In, Bal, and Out players, ∆*Ni*(*t*), where *k* denotes a time lag. The results are given

−

in Tables 5 and 6. There are correlations between *k* = 0 and *k* = 1 in both BTC and XRP for active players, which implies the price changes follow the changes in the number of users in this target period. In contrast, it turns out that there is no correlations for regular players. In addition, we examined other different periods that cover several crypto bubbles and crashes (see Figures 9 and 10). The results for the correlations for these periods are summarized in Table 7.

−

3. *Is it possible to make any price prediction based on the number of active players or rations between various players?*

## response

We conducted a VAR (vector auto regressive) analysis to examine the possibility of predicting price as the reviewer commented as an interesting point. See the Supporting Information’s section "Granger Causality Analysis of Price and Number of Players" for details. In a nutshell, the results show that the number of players *xt* is not the cause of price *yt* in the Granger sense. Including *xt* in the model does not improve the prediction accuracy of *yt*. On the other hand, *xt* can be an instantaneous cause of *yt*. However, such instantaneous causality does not usually help the prediction for an obvious reason. These results indicate that it would be difficult to forecast the prices of cryptoasset using the number of players.

4. *"The ratio of Bal-players is larger before the peak, but after the peak, the ratio of In-players is larger." It is quite curious that there were more players after the peak and the price was sharply dropping?*

## response

This behavior looks so interesting as the reviewer pointed out. This can be considered to be characteristic to the regular players. In the bubble period, there appears to be a change in position from Bal to In. Regular players are presumably large financial institutions such as exchanges. They do not stop their business and would not exit the network of crypto flow even during the bubble collapse; instead, they could change their positions between In, Bal, and Out. In our additional analysis for other periods (see Figures 9 and 10), we found that even during the big bubble period in 2020, there exists a slight change in position from Bal to In in the regular players.

5. *"This fact suggests an abrupt change in the number of Players at the price peak seen in the upper panel of Fig. 4 for BTC regular players are due to a change in the number of non-identified regular players" this may be the effect of the influx of new players encouraged by the bull market?*

## response

Precisely. We meant by the sentence that this may be related to the entry of new players encouraged by the bull market. We added a sentence to convey this viewpoint in the revised manuscript.

6. *Line 203 P^XRP32 not P^BTC32*

## response

We corrected the typo. We thank again for the reviewer’s help.
